# Machine learning and artificial intelligence: Enabling the clinical translation of atomic force microscopy-based biomarkers for cancer diagnosis

**DOI:** 10.1016/j.csbj.2024.10.006

**Published:** 2024-10-05

**Authors:** Aidan T. O’Dowling, Brian J. Rodriguez, Tom K. Gallagher, Stephen D. Thorpe

**Affiliations:** a UCD School of Medicine, University College Dublin, Dublin, Ireland; b UCD Conway Institute of Biomolecular and Biomedical Research, University College Dublin, Dublin, Ireland; c Department of Hepatobiliary and Transplant Surgery, St Vincent’s University Hospital, Dublin, Ireland; d UCD School of Physics, University College Dublin, Dublin, Ireland; e Trinity Centre for Bioengineering, Trinity College Dublin, Dublin, Ireland

**Keywords:** Force spectroscopy, Tissue mechanics, Cell mechanics, Mechanobiology, Biophysics, Atomic force microscopy

## Abstract

The influence of biomechanics on cell function has become increasingly defined over recent years. Biomechanical changes are known to affect oncogenesis; however, these effects are not yet fully understood. Atomic force microscopy (AFM) is the gold standard method for measuring tissue mechanics on the micro- or nano-scale. Due to its complexity, however, AFM has yet to become integrated in routine clinical diagnosis. Artificial intelligence (AI) and machine learning (ML) have the potential to make AFM more accessible, principally through automation of analysis. In this review, AFM and its use for the assessment of cell and tissue mechanics in cancer is described. Research relating to the application of artificial intelligence and machine learning in the analysis of AFM topography and force spectroscopy of cancer tissue and cells are reviewed. The application of machine learning and artificial intelligence to AFM has the potential to enable the widespread use of nanoscale morphologic and biomechanical features as diagnostic and prognostic biomarkers in cancer treatment.

## Graphical Abstract

**Figure:**
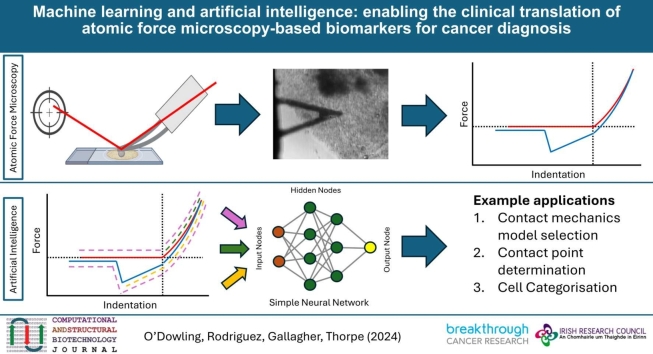

## 1 Introduction

Biomechanical signalling is known to play a critical role in normal cellular processes from embryology to apoptosis [1]. Mechanotransduction, the ability for cells to convert mechanical forces into biochemical pathways, is an integral part of the normal functioning of cells [2]. Furthermore, dysregulated mechanotransduction appears to play a vital role in oncogenesis, for example through the transcriptional co-activators YAP and TAZ which are activated by biomechanical stimuli [3]. These proteins have been shown to be crucial in maintaining cancer stemness [4]. The extra-cellular matrix (ECM) is the support network around cells, which plays a vital role in basic cellular functions, forms the microenvironment, and provides structure [5]. Solid cancers are often associated with increased tissue stiffness, and this increased stiffness has been shown to drive cancer progression [6], [7], [8]. Furthermore, increased ECM stiffness has been shown to induce malignant phenotypes in normal cells in vitro [9].

Cell and tissue biomechanics can be assessed on various length scales in tension, compression and shear using a variety of approaches. Given the role of ECM stiffness and solid stress in supporting cancer cell phenotypes and signalling associated with invasion and metastatic spread [8], [10], [11], [12], understanding tissue biomechanics on the cellular length scale is of paramount importance to generating biomechanical biomarkers to diagnose or predict prognosis in solid tumours [13]. Perhaps the best-established method for assessing biomechanical properties at the cell length scale is atomic force microscopy (AFM) force spectroscopy. AFM facilitates assessment of stiffness, topography and adhesion at nm length scales relevant to structures such as subcellular cell-ECM adhesions, the cytoskeleton and cell membrane, or μm length scales relevant to whole cell or ECM properties [14].

Despite the clear potential of AFM-based biomarkers as diagnostic and prognostic indicators, this approach has not been adopted clinically as yet. Factors preventing the clinical adoption of AFM include the relatively high system cost, although system design is improving such that clinically targeted systems can be built more economically than multi-purpose research systems with greatly simplified experimental setups as demonstrated by companies like Artidis (Basel, Switzerland) [15]. Perhaps the most significant impediment to clinical adoption is the high cost in terms of training time required to develop the technical expertise to analyse AFM datasets. Here artificial intelligence (AI) and machine learning (ML) can provide solutions: by expediting the AFM measurement process, by automating analysis, and by classifying cells or tissues based on derived or established nanomechanical biomarkers. This review aims to summarise the current state of the art in the application of AI or ML toward AFM application in the field of cancer, and identify how AI and ML could enable the future clinical translation of AFM-based biomarkers for cancer diagnosis.

## 2 Atomic force microscopy for cell and tissue mechanics

**Fig. 1:**
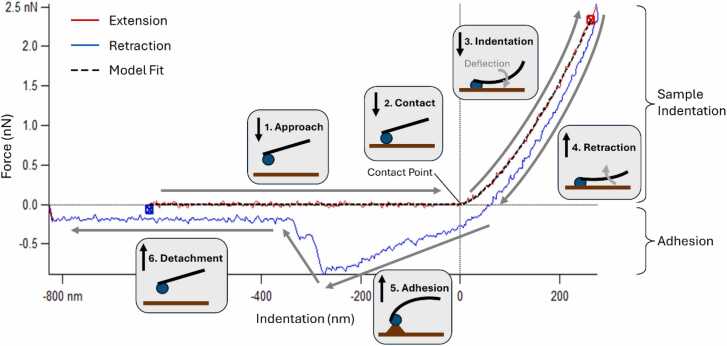
Example force – indentation curve obtained using AFM in force spectroscopy mode. Direction of probe travel in relation to the sample is overlaid with schematic representation of a colloidal probe – specimen contact sequence. 1. Approaching contact. 2. Contact point. 3. Sample indentation and cantilever deflection. 4. Probe retraction with sample hysteresis. 5. Adhesion of probe to sample generating a negative force on retraction. 6. Probe detachment from the sample. Alternative probe geometries such as pyramid or cone would utilise the same sample contact sequence.

AFM was first described as a high resolution scanning probe microscope in 1986 [16]. A tip at the end of a typically vibrating cantilever comes into contact with a specimen (Fig. 1). Both long and short range attractive and repulsive forces act on the tip leading to deflection of the cantilever. This deflection can be used to calculate sample topography. In attempting to overcome these interactive forces, in particular van der Waal’s forces, it was discovered that in addition to imaging materials at the nanoscale level, AFM could also be used to determine mechanical properties by investigating the forces between the sample and the tip [17]. Force-indentation curves can now be easily obtained using the force spectroscopy modes of commercial AFMs [18]. Force measurements are obtained using Hooke’s Law: *F = k*_*c*_ *.d;* where *F* is force [N], k_c_ is the cantilever spring constant [N/m] and d is the cantilever deflection [m].

Force spectroscopy techniques have continued to evolve over the past three decades. Measurements were originally performed in air, but in the mid-1990s it was shown that performing analysis in liquid reduced van der Waal’s forces and eliminated capillary forces [19], [20]. More recently, advanced methods such as peak force tapping [21], off resonance tapping mode [22], and high frequency rheology [23] can offer scan rates up to 50 frames per second [24], [25]. Different high speed nanomechanical mapping methods have been tested in several studies and most have been shown to be consistently accurate when compared to traditional modes [26], [27]. Perhaps the most commonly employed high speed AFM mode is Peak Force Tapping which offers synchronous topographical and indentation measurements at a much faster rate than standard modes. Introduced in 2011, it has become increasingly used due to its speed. By employing a constant feedback loop, a sinusoidal motion of the tip, and no force at its contact point, it operates at rates up to 8 kHz [21], [28], [29]. Rather than a feedback loop based on cantilever deflection amplitude, this mode maintains a low peak contact force between the tip and the sample through sinusoidal tip movement. However, due to low contact force and indentation depths, mechanical measurements are not recommended in this mode, and it is most relevant to imaging. Peakforce Quantitative Nanoscale Mechanical Characterisation (QNM) has been developed to provide mechanical measurements, although biological materials are softer by several orders of magnitude than the lower end of the recommended range for this mode; 1 MPa – 100 GPa for Peakforce QNM compared to 10 Pa – 10 kPa for most human tissues. Modifications to tips, cantilevers, and stages have been described to improve high speed force measurements, prevent surface drag damage, and accurately focus on the field being investigated, but no universal standards have been adopted [25]. Fast nanomechanical mapping modes provide vast quantities of data, e.g. by enabling high resolution scanning of surface features and topography, but this can make analysis challenging and time consuming.

Tip geometry plays an important role in measurements and affects how forces are calculated. Due to the precise technical nature of AFM experiments, any inter-operator differences in execution can result in significant variations in results. Some studies have shown that conical probes provide stiffnesses values two to three times greater than colloidal (spherical) probes [30], [31]. Biological samples are often heterogeneous on the micro-scale and colloidal probes are often used to assess tissue and cell mechanics. By varying the colloidal probe size, a global view of a specimen’s mechanical properties can be obtained, averaging out spatial heterogeneity. This, in turn, reduces the need for many measurements over a small area, as obtained using high-speed AFM approaches such as the Peakforce mode described above. A faster movement and set up from one area of the specimen to the next distant area to be measured is more relevant for this type of analysis and is currently not available in the high output modes offered. Certain ML algorithms have automated movement of the AFM tip between regions, as described below, but this is not yet available commercially [32], [33]. The spherical shape also simplifies model choice; the Hertz model provides the most commonly used model in the analysis of force-displacement curves to obtain the elastic modulus [17]. Spherical tips are preferred as they offer a more well-defined geometry with less risk of unknown damage to the tip [34], damage due to plastic deformation [35], or inadvertently piercing the specimen with a sharp tip [36]. Pyramidal or conical indenters have sharp tips which provide topography images and enable force measurements at nanometre spatial resolution. However they wear more quickly and can become damaged through routine use [34]. Damaged or worn tips are a constant challenge as they must be recognised to avoid recording inaccurate results [37]. The Sneddon-modified Hertz model is most often used to fit force spectroscopy curves using pyramidal or conical probes [38].

The Hertz model is the oldest and simplest model for contact mechanics but carries multiple assumptions, including that surfaces are continuous, the sample can be considered an elastic half-space, and perhaps most relevant to biological samples, surfaces are free of adhesion [17], [38]. Two other models, the Derjaguin-Muller-Toporov (DMT) and Johnson-Kendall-Roberts (JKR) models account for adhesive forces of different kinds [39], [40]. DMT addresses issues with the effective normal load, i.e. weak or long range forces (e.g. van der Waal’s forces) [39]. JKR is more relevant to biological samples. It accounts for strong adhesion and short range interactions which cause an increase in the effective contact area; typical of cells and tissues [40]. However, there are no precise cut off points where each model is appropriate, and factors in addition to adhesion also drive this decision. For example, as load increases, the ideal model for all scenarios, regardless of adhesion, approaches Hertz [41]. Despite these considerations, Hertz remains the most commonly used model in AFM experiments of biological materials.

Several others have recently reviewed the use of AFM in cancer research with a focus on cell [42], [43] or tissue mechanics [44]. While these illustrate the potential of AFM based nanomechanical biomarkers in the study and diagnosis of cancer, they do not address the issues with translational of AFM from a research to clinical setting. The cost of a machine generally ranges from $100,000 – $500,000 and it takes weeks to months to train someone to be proficient in both the technical and analytical procedures involved. AFM systems typically require installation in a quiet and vibration free environment, on an anti-vibration stage within an acoustic chamber. This adds a challenge to locating traditional AFM systems in busy clinical settings, although these physical challenges are not addressed in this review. Here we outline how artificial intelligence (AI) and machine learning (ML) techniques have been employed with AFM in cancer research to date, and discuss how AI and ML could enable clinical translation in the future.

## 3 AFM based nanomechanics in cancer research

### 3.1 Tissue mechanics in solid tumours

For as long medicine has been practiced, firm, palpable growths have been associated with cancer. However, we now know that for many solid cancers, tumours and adjacent normal tissue from which the cancer is derived are biomechanically distinct in two key aspects. One mechanical change in tumour tissue is an increase in Young’s modulus [7], [13], [45]. This is a result of the upregulated production of collagen and other ECM components [46], [47]. AFM has been used to document this increase in tissue stiffness in numerous cancers [44]. However, a common observation in tumours, first described by Plodinec *et al.* in breast tissue, is the increased heterogeneity in mechanical properties within the tissue relative to healthy controls [48], [49], [50]. This manifests as a bi-modal distribution in mechanical property values across a tumour section, with a soft peak associated with cancer cells and a stiffer peak and tail associated with stiff stromal extracellular matrix [51].

Stiffer matrices are associated with both increased cancer cell stemness [4], [52], and increased metastatic potential [53], [54]. However, while tumour tissues are considered stiff, aggressive cancer cells are often soft [13], [45]. *In vivo,* stiffening of the extracellular matrix has been hypothesised by Lorenc *et al.* to be a nidus for metastatic spread, as shown in the peritoneal metastases of colorectal cancer [55].

While tissue stiffness measurements are typically conducted on fresh tissue or defrosted fresh-frozen tissue sections, some have used formalin fixed paraffin embedded (FFPE) tissue for AFM assessment [56]. While this results in greatly exaggerated stiffness values, it remains possible to discriminate between tumour and healthy tissues, with tumour heterogeneity a factor in both stiffness and topography maps of FFPE sections of healthy skin, benign naevi and melanoma [56].

### 3.2 Cellular mechanics in solid tumours

Cancer cells differ structurally to healthy cells, providing the basis of histopathological examination and diagnosis. These structural changes result in altered cell mechanics. As indicated above, cancer cells are generally softer than their fully differentiated, non-cancerous counterparts. This was first demonstrated in 1999 by Lekka *et al*. who used AFM to differentiate between normal and cancerous bladder cell lines, showing that normal cells were significantly stiffer than tumour cells [57]. This has further been shown in other cancers including lung [58], breast [58], oesophageal [59], pancreatic [58], [60], and prostate [61]. Rianna *et al.* demonstrated that tumour cells get softer as their surroundings become increasingly confined [62]. This gives further support to the theory that metastatic cells must have the ability to deform [63], and supports the results of Chen *et al.* who demonstrated in a murine model that breast cancer metastases to bone are much softer than the subcutaneous primary tumour [64].

High grade tumour cells or cells with a higher metastatic potential have been shown to have lower elastic moduli than their lower grade equivalents [65], [66], [67], [68]. However, all tumour types do not necessarily behave this way with Daniel *et al.* demonstrating that chondrosarcoma cells are stiffer than healthy chondrocytes [69]. The increased compliance in cancer cells may have a functional role. Wang *et al.* used AFM to mechanically stimulate normal MCF10A and cancerous MCF7 breast cells every 30 s for 1 h, and observed that cancer cells survive while normal cells detached, indicating that the cancer cells could better endure repeated mechanical deformation [70].

Cancer cell stiffness in 2D culture conditions tends to conform to the substrate on which cells are cultured, with cells cultured on stiff substrates, mimicking tumour stroma, exhibiting higher elastic moduli than cells cultured on softer substrates [4], [71], [72], [73]. Substrate stiffness also modulates cell morphology, with breast cancer cells cultured on soft substrates exhibiting lower spread area, a more circular shape, and increased height than those cultured on stiff ECM [71]. This is likely a result of increased cytoskeletal tension on stiff substrates in 2D. However, 2D observations do not necessarily translate to 3D scenarios.

Cell mechanics are often assessed in the context of cell morphology and cytoskeletal structure. Changes to nanoscale morphology not visible at the macro or microscales have been described using AFM. For example, myofibril regularity and nucleus size can be used to differentiate between normal uterine tissue, benign tumours, and leiomyosarcomas [74]. Morphological differences between normal and cancerous cells have also been shown using AFM in liver cancer [75]. Ezenwafor *et al.* visualised individual actin filaments using AFM in different grades of breast cancer, where higher grades were associated with decreases in both actin filament alignment and density [76]. These can all be used as an aid to diagnosis and could help distinguish borderline cases or where traditional diagnostics are deemed inconclusive.

Although the most commonly reported, elasticity is not the only biophysical property measured by AFM. Both adhesion and viscoelasticity are commonly investigated in the biomechanical assessment of cancer cells and tissues. Adhesion is a regulator of tumour cell migration, invasion, and metastasis [77]. High grade glioma cells have been shown to exhibit lower adhesive forces than cells from low grade gliomas, illustrating this point [78]. Following a similar pattern to cell mechanics, increased adhesion is seen in cells cultured on stiffer substrates [72]. Viscosity demonstrates a similar disease profile to other mechanical properties, particularly the elastic modulus, with concurrent increases and decreases seen in several diseases including breast cancer [76]. Viscoelasticity has also been associated with ovarian cancer cell invasion [79]. Varga *et al.* meanwhile compared the adhesive properties of different melanoma cell lines to brain endothelial membrane by fixing melanoma cells to tipless cantilevers [80]. The non-metastatic cell line demonstrated the lowest adhesion to the membrane which indicates that adhesion of tumour cells could directly affect metastatic potential. These results indicate that elasticity is not the only biomechanical property which should be routinely measured, and other viscoelastic properties may also have a role in diagnosis and prognosis.

### 3.3 Extracellular vesicle mechanics

Exosomes are extracellular vesicles formed by all cells and can be identified in liquid biopsies. These are associated with cancer progression and metastasis and can provide valuable liquid-based biomarkers [81]. AFM has been used to visualise individual exosomes [82] and compare the structural and biomechanical properties of exosomes derived from different tissues. Indeed, Feng *et al.* have shown that the elastic and viscous properties of extracellular vesicles derived from multiple myeloma patients differ from EVs isolated from healthy volunteers [83]. Yurtsever *et al.* showed that exosomes derived from both aggressive and non-aggressive osteosarcoma cell lines have similar structural appearances and sizes, but distinct elastic moduli [84]. Further work is required to understand the functional effect of EV stiffness and whether this plays a role in targeting tissues for metastatic priming.

### 3.4 Dynamic cell and tissue response to stimuli

High-speed AFM is increasingly used to measure the effects of stimuli, e.g. extracellular vesicle addition, on tumour or stromal cell behaviour. AFM has been used to characterise normal, cancerous, and metastatic liver cell lines treated with metastatic hepatocellular carcinoma-derived exosomes. These cells exhibited a reduction in their elastic modulus and adhesion properties in addition to morphological changes in response to treatment [85]. Similar cell morphological and biomechanical changes in response to cancer cell-derived extracellular vesicles have also been shown in non-small cell lung cancer [86]. As described above, low stiffness and adhesion is characteristic of higher metastatic potential, indicating that exosomes derived from aggressive tumours can induce and perpetuate further aggressive phenotypes.

Chemotherapeutic medications such as cetuximab and ROCK inhibitors administered to breast cancer cell lines and echinomycin in ovarian cancer cell lines have been shown to induce cancer cell stiffening [71], [79], [87]. Similar results have been shown for hepatocellular carcinoma, and non-small cell lung cancer in response to sorafenib tosylate and osimertinib mesylate respectively [88]. We have previously shown that pancreatic cancer cell stiffness can be reduced through targeting of the retinoic acid receptor-β pathway [89], or G protein-coupled estrogen receptor [90] leading to a reduction in metastatic phenotype. Doxorubicin has also been shown to cause a reduction in elastic modulus in a variety of cell lines [72] as well as in a series of patient tissue samples [45]. Doxorubicin is known to induce cytoskeletal remodelling which is likely the cause of such results [91]. Cisplatin and paclitaxel have been shown to stiffen melanoma cells, possibly due to an increase in microtubule stability [92]. In addition to mechanics, AFM also captures nanotopography and cell morphology, and can identify impairment of cellular structure or an increase in roughness; both of which can indicate apoptosis [88], [93], [94], [95]. Liu *et al.* observed increases in both height and stiffness of the circulating tumour cells of patients undergoing chemotherapy for ovarian cancer [96]. In general, the effects can be measured to evaluate dose-dependent changes [88], [96], [97] and in some cases have demonstrated that aggressive cell lines have more profound responses to treatment than their less aggressive counterparts [98]. Analysis of cellular nano-motion, i.e. vibrations induced by cells adhered to a tipless cantilever, has been proposed as a measure of cell viability [99] and cytoskeletal modulation [28]. This approach has been demonstrated to provide a measure of multidrug resistance in cells from several cancer types treated with doxorubicin [100]. Epithelial to mesenchymal transition (EMT), a process associated with cancer invasion, progression, and metastasis, has also been studied via AFM. Liu *et al.* treated colorectal cancer cells with tumour necrosis factor-α to induce EMT and observed both increased incidence of stress fibres and reduced cellular stiffness [101].

Recent advances in AFM have made both the acquisition and contextualisation of results easier. Most, however, have yet to be used in the setting of oncology research. Dynamic mechanical analysis involves cyclical frequencies being applied to a specimen with the specimen’s strain being measured to determine a number of viscoelastic properties [102]. Standard AFM results are typically calculated using the first order resonant frequency of the cantilever being used. Higher order resonant frequencies (or harmonics) are also present and multi-harmonic AFM examines multiple frequencies simultaneously for accurate and fast nanomechanical characterisation [103]. This approach was further improved upon by speeding up the acquisition of data [104]. New approaches to assessing biomechanical properties using AFM are regularly described, although the application of these novel approaches requires experienced AFM users to apply them to biology and oncology.

## 4 Application of ML to AFM in cancer research

Although colloquially used interchangeably, artificial intelligence (AI) and machine learning (ML) do not describe the same concept. AI, first described by McCarthy in 1955 [105], [106], is a broad term but at its most fundamental is the ability for computers to perform tasks traditionally associated with intelligent beings. ML is a subdivision of AI; computers solve problems via algorithms based on pattern recognition. While some aspects of AI are based exclusively on logic-based solutions (symbolic artificial intelligence), ML leverages statistics and probability.

The recent boom in artificial intelligence has led to a marked increase in the number of domains employing machine learning and neural networks, a branch of AI, and the fields of cancer and AFM have been no different. ML is actively being pursued in clinical diagnosis and prognosis, where the ML approaches are used to identify patterns in patient data and/or omics and image datasets. The use of AFM in oncology remains relatively novel, and so the application of AI in the context of AFM is in its infancy. To date, several AFM studies of cancer cells have used artificial intelligence in their analysis. ML alongside AFM has been applied to several different cancer types, including breast, bladder, colorectal, and lung with multiple different models, and a variety of inputs and outputs investigated [107], [108], [109], [110]. To date, the most broadly accepted goal of ML in AFM is to speed up analysis. There are several disadvantages to AFM which hinder its use clinically. These include time consuming sample preparation and setup, the high cost of purchasing an AFM, slow speed of scanning relative to other approaches such as light microscopy, and the time required for a user to both learn the technique and analyse the data. Although some aspects, like sample preparation, cannot be immediately improved by ML, there is a clear opportunity for ML to augment analysis.

### 4.1 Machine learning models

Many different methods of ML have been used in the field of AFM. More traditional models include support vector machines (SVMs) and random forest (RF) algorithms [108], [111]. SVMs are supervised learning models for classification and regression. RFs, meanwhile, involve the creation of many decision trees (often thousands), using randomly observed features of random datapoints. While it is possible to interrogate each individual tree, it would often be overwhelming to analyse each individually, limiting the transparency of this approach. However, the ML methods most commonly used in the field of AFM are a family known as artificial neural networks (ANNs), often shortened simply to neural networks (NNs) [107], [109], [110], [112], [113], [114], [115]. These are amongst the most powerful ML models. Akin to the biological brain, these have multiple interconnected nodes (artificial neurons), through which information can be passed from one to another. Data are entered into the input nodes, transferred and analysed through a series of hidden nodes, and finally a result is expressed through the output node(s). Although these provide the most powerful ML models, a significant limitation of NNs is their complexity. Unlike other models where the developer decides where decisions will be made, NNs don’t naturally explain how a decision was arrived at; whereas RFs are considered “grey box” due to their opacity to the observer, NNs are often considered “black box” due to their closed off nature. While it is possible to access each node, it can be challenging.

Nguyen *et al.* tested several ML algorithms to automate use of the most relevant contact mechanics model for AFM curve fitting [116]. Basic algorithms such as simple decision trees and k-nearest neighbours were assessed, but as expected, more complex models function to a higher standard, in this case linear discriminant analysis. The conflict between model transparency or explainability and complexity continues to persist and is particularly relevant to the clinical adoption of such an approach. How these models have been applied to AFM is appraised in Section 4.3 below.

### 4.2 Model evaluation

ML models are assessed in several ways. A crucial step in creating a model is internal validation of the algorithm, known as the train/test split. Here part of data available for training is withheld, and instead used to validate the tool. Overfitting occurs when the algorithm created has too many elements from the training data such that the model will only work for that dataset and will not accurately predict other data. Loss versus epoch plots are a useful tool to assess training progress and model overfitting. For example, Hui *et al.* use this approach to compare the training efficiency of their long short-term memory (LSTM) based recurrent neural network learning model to alternative approaches [110]. However, this approach does not provide information on model precision or accuracy.

The receiver operating characteristic (ROC) curve is widely used in medical research to evaluate the accuracy of diagnostic tools, and it is also the most widely used tool in evaluating ML in this context. It plots true positives (sensitivity) against false positives (1 – specificity). The area under the curve (AUC) can then be derived from this graph. AUC is a value which can range from 0 to 1, where a value of 1 provides the ideal diagnostic tool. A similar approach is the precision-recall curve which plots true positives as a fraction of predicted positives (precision) against the number of true positives as a fraction of actual positives (recall). This is useful for unbalanced datasets, for example assessing the accuracy of Rade *et al.*’s ML model in detecting cell shape [33].

Clinical adoption of ML approaches will require model evaluation using tools such as these, perhaps in addition to the evaluation of the provided diagnostic tool which may include non-ML elements. At present, there are no established criteria for model evaluation, unlike traditional clinical diagnostics, and of the studies applying ML to AFM described in Section 4.3 below, some do not describe model validation [109], [113]. While guidelines do exist for developing, evaluating, and reporting ML models in biomedical research [117], field-specific and publisher backed consensus is required to ensure reproducible application [118].

### 4.3 Use of ML in AFM analysis

A variety of cancer types have had ML methods applied to AFM investigations, including bladder, breast, colorectal, cervical, lung, and brain [107], [108], [109], [110], [111], [112], [113], [114], [115]. These are summarised in Table 1. These studies have applied ML approaches to cancer diagnosis or prognosis through the automated identification and/or characterisation of cancer cells or tissues.

**Table 1:** Application of ML and AFM as biomarkers of cancer diagnosis or prognosis.

Reference	Application	Results	AFM Approach	AI Model
Ciasca *et al.*[114]	Automation of force-indentation curve analysis to categorise resected brain tissue into tumour or healthy categories. See also Minelli *et al.*[115].	Fewer force-indentation curves are required to categorise tissue when using NN based assessment of indentation curves.	JPK Nanowizard II AFM (Veeco) with a Zeiss AxioObserver; Uncoated, silicon cantilevers with pyramidal tip	Feed-forward neural network
Hui *et al.*[110]	Detection of mesenchymal-to-epithelial transition (MET) on invasive non-small cell lung cancer cells by measuring the range of oscillations of the cell surface over time	Surface undulations of cells undergoing MET have two distinct peaks. Lung cancer cells could be identified with > 90 % accuracy and circulating tumour cells with > 80 % accuracy based on these undulations	JPK Nanowizard II AFM with a Zeiss AxioObserver and fluid-cell mounted cantilever (Microlevers, Veeco); 200 nm bovine serum albumin-coated polystyrene tip held in position for 4 h on sample and undulations recorded	Long short-term memory recurrent neural network
Minelli *et al.*[115]	Automation of force-indentation curve analysis to categorise resected brain tissue into tumour or healthy categories. See also Ciasca *et al.*[114]	Modulus could be used to diagnose glioblastoma and meningothelial meningioma tumour tissue.	JPK Nanowizard II AFM (Veeco) with a Zeiss AxioObserver; Uncoated, silicon cantilevers with pyramidal tip	Feed-forward neural network
Petrov and Sokolov[109]	Identifying morphological patterns associated with aggressiveness in colorectal cancer cell lines	No individual feature examined can fully explain why a region is highly vs less aggressive	Ringing Mode (AFM model not provided)	Gaussian process regression
Petrov and Sokolov[119]	Differentiation of precancerous and cancerous cervical cells through topographical analysis of AFM adhesion maps	Precancerous and cancerous cervical cells can be distinguished effectively with an ROC-AUC of 0.93 %, and 83 % accuracy. Sensitivity is a major shortcoming of current diagnostics and a value of 92 % was achieved.	Nanoscope™ Dimension 3100 AFM (Veeco/Bruker-Nano) with Nanoscope V; HarmoniX (subresonance tapping) mode	Random forest
Sokolov *et al*.[108]	Identifying bladder cancer cells via surface topography features from patient urine	Diagnosed bladder cancer through analysis of cell surface characteristics in urine with diagnostic accuracy of 94 %, which is significantly better than the current clinical standard.	Bioscope Catalyst (Bruker/Veeco) AFM with Nanoscope V controller; PeakForce Tapping (0.1 Hz); Ringing mode (0.4 Hz); Bruker ScanAssyst cantilevers; in air	Random forest, extremely randomised forest, gradient boosting trees
Wang *et al*.[111]	Identification of malignant cells from lung, cervical, and breast cell populations by topography, prestress, and elastic modulus	In all normal and cancerous cell lines tested, cancer cells have lower modulus and higher prestress. The algorithm has a classification accuracy of 89 % and ROC-AUC of 0.93, with a signal-to-noise ratio 8 times that of human cytologist-based morphological analysis approaches shown in a single lung cancer patient.	NX10 AFM (Park Systems); Gold-coated, rectangular, silicon cantilevers with pyramidal tip, mounted in liquid	Support vector machine
Weber *et al.*[113]	Identification of treatment populations in breast cancer cells by viscoelastic cell mechanical properties	Moderate ability to differentiate between breast cancer cells that underwent different treatments.	JPK Nanowizard III with CellHesion extension; gold-coated silicon nitride pyramidal probes	Self-organising (Kohonen) maps
Zeng *et al.*[121]	Identification of hepatocellular carcinoma cells from hepatocytes by cell morphology and mechanics datasets.	Classification accuracy of 94.5 % and ROC-AUC of 0.99 was achieved with the GaussianNB algorithm which performed best.	JPK Nanowizard III; SHOCONG−10 probe with pyramidal tip	GaussianNB algorithm; later compared to logistic regression and Support vector machine
Zhu *et al.*[112]	Grading of bladder cancer stage in cell lines using elastic modulus, cell membrane tension, adhesion, and work of adhesion	Graded bladder cancer cell stage with an accuracy of 91.25 % and ROC-AUC value of 0.9798.	JPK Nanowizard III mounted on an inverted microscope (Olympus) with spherical tipped probe	Back propagation neural network

Force spectroscopy has been widely used to demonstrate differences in cell and tissue mechanics in cancer. However, single metrics such as elastic modulus are limited as diagnostic or prognostic tools due to high inter and intra sample variability. Weber *et al.* address this by deriving several viscoelastic parameters from each AFM curve which are then used to train unsupervised self-organising maps [113]. This approach was able to discriminate between treated and untreated breast cancer cells while the obtained maps support investigations of the relationships between treatment variables.

Surface topography is another dataset obtained via AFM. Sokolov *et al.* used subresonance tapping mode to image the surface topography of relatively small numbers of fixed cells collected from urine and were able to diagnose bladder cancer with an accuracy of 94 % [108]. This is both less invasive and more accurate than the current clinical standard of cystoscopy. Notably, ML was not applied to the topography images, but to a set of engineering surface parameters which reduced the dimensionality of the dataspace. This group has also demonstrated that a modified version of this approach can discriminate between colorectal cancer cell lines with high and low neoplastic aggression, although this has yet to be translated to clinical colorectal cancer samples [109]. By applying this same surface parameterisation approach to AFM adhesion maps, Petrov and Sokolov have demonstrated good precision in distinguishing pre-cancerous and cancerous cervical cancer cells, with an ROC-AUC of 0.93, but notably a sensitivity of 92 % which is a stark improvement on current colposcopy tests [119].

Another study in bladder cancer used a NN to discriminate between cells in four stages of cancer initiation/progression based on a set of four cellular mechanical properties: elastic modulus, membrane tension, adhesion and work of adhesion [112]. With this approach Zhu *et al.* were able to obtain an ROC-AUC value of 0.9798 which surpasses current diagnostic approaches [108], [120], although these studies were conducted using cell lines and have yet to be translated to patient cell isolates in the clinic.

Combining multimodal AFM datasets in this manner can be advantageous as single parameters are not typically predictive [109]. Zeng *et al.* combined measures of subcellular topography, adhesion and elastic modulus using the GaussianNB algorithm to successfully discriminate hepatocellular cancer cells from hepatocytes with an accuracy of 94 % and ROC-AUC of 0.99 [121]. Wang *et al.* created a “mechanome” based biomarker consisting of AFM line scans across each cell and found that training a SVM model using corrected modulus and prestress mechanome profiles supported the identification of precancerous cells from normal cells with a signal-to-noise ratio eight times that of human cytologist based morphology assessment [111].

An alternative approach is to measure cellular perturbation of the AFM probe, i.e. cell membrane undulation, over an extended duration of contact. Hui *et al.* used this approach to predict whether non-small cell lung cancer cells had undergone mesenchymal to epithelial transition, important for metastatic spread. To do this, they implemented a NN which could broadly diagnose cancerous cells by analysing cell membrane undulation spectra [110]. Two distinct frequency peaks of cell membrane undulation were identified in cells after undergoing mesenchymal-to-epithelial transformation. Based on this, the algorithm could accurately identify > 90 % of lung cancer cells and > 80 % of related circulating tumour cells.

The studies described above used metrics derived from AFM topography, stiffness, or adhesion maps to identify cancer cells or define cell phenotype. An alternative approach is to use ML to assess the obtained force curve negating the need for curve fitting. Minelli *et al.* used a NN to analyse force-indentation curves and could discriminate between brain tumour and benign tissue [115]. This group has since expanded on this approach to also reduce AFM measurement time by estimating the minimum number of force curves required [114].

AI also has potential in speeding up AFM result acquisition by reducing both the time required to collect and analyse datasets, and the costs traditionally associated with training humans to perform AFM and analyse the data. Fitting force-distance curves requires a specialised skillset, can be arbitrary, and is time-consuming. There have been attempts to automate force curve analysis in the past, for example FC_analysis and OpenFovea [122], [123], although none have become commonplace. In-built AFM software systems have improved to speed up manual analysis and are often considered more reliable. However, ML has made it possible to automate this and remove user bias while obtaining accurate identification of cancerous and non-cancerous tissue [115]. Studies applying ML toward expediting AFM analysis processes are described in Table 2.

**Table 2:** Application of ML to expedite AFM nano-mechanical analysis.

Reference	Application	Results	AFM Approach	AI Model
Nguyen and Liu[116]	Automation of contact model selection based on AFM indentation curve. Compared five machine learning models	Automatically employed most appropriate contact model and found modulus without pre-processing knowledge of tip shape to accuracy of 96.8 %. Of the models tested, LDA was the most accurate model as per the train/test split.	Icon Dimension AFM (Bruker); PeakForce mode; Silicon cantilevers with conical tips	Decision tree, K-nearest neighbours, linear discriminant analysis (LDA), naïve Bayes, multiclass support vector machine
Nguyen and Liu[124]	Determination of sample elastic modulus without contact model fitting or knowledge of tip shape. Compared four machine learning models	Automatically determined elastic modulus of both homogeneous and heterogeneous materials with accuracy of 91.5 % and 82.7 % respectively. Of the models tested, GPR had the best R^2^ value.	Icon Dimension AFM (Bruker); PeakForce and Force Volume modes; Silicon cantilevers with pyramidal tips	Gaussian process regression (GPR), multiple linear regression, random forest, support vector machine
Rade *et al.*[33]	Identification of cells for analysis based on light-microscopy morphology, and subsequent optimisation of AFM navigation using machine learning. AFM based nano-mechanical analysis was conducted using standard approaches.	60x reduction in time required to move from one cell to another for force-indentation curve acquisition	BioResolve AFM (Bruker); PeakForce QNM mode	Convolutional neural network
Sotres *et al.*[126]	Location of the contact point in an AFM force-indentation curve	Accurately identified contact point. Curve complexity increased the time required to train the model	MultiMode 8 SPM NanoScope V (Bruker); Silicon nitride cantilevers with pyramidal tip	Convolutional neural network

Nguyen and Liu first used linear discriminant analysis to develop an algorithm which recommended the most appropriate contact mechanics model with which to fit the data [116], and then tested multiple ML models to find an algorithm which projected mechanical properties based on force-indentation curves with no contact mechanics model applied [124]. A gaussian process regression model predicted the elastic modulus of homogeneous materials with 91.5 % accuracy. Kamble *et al.* developed a NN with a 97.5 % accuracy rate of predicting mechanical properties based on cell morphology and raw force-indentation curves, although this has yet to be applied to cancer cells [125].

A further potential process for significant acceleration of analysis includes automated identification of specific events in a force-indentation curve. Identifying the contact point is another example of a necessary and time-consuming task which can be automated, as demonstrated using a NN by Sotres *et al.* [126]. Another feature of AFM force spectroscopy curves are rupture events between the probe and bound molecules/cells which are typically manually identified and can be numerous. Waite *et al.* implemented a deep learning NN to identify cell membrane rupture events in force-spectroscopy curves which could match the performance of moderately trained humans [127]. Perhaps the most radical use of ML and AFM to date has been to train a ML model using tissue mechanics data to subsequently estimate Young’s moduli of tissue samples based on collagen organisation. This method was used to estimate the stiffness of tissues which had been formalin fixed [107].

ML algorithms are also applied to speeding up the AFM force curve acquisition process. Two separate studies have applied NN to the identification of cells based on their appearance under light microscopy, which then instructed the AFM stage to move to the cell types chosen in advance [32], [33]. This significantly increased the speed of force-indentation curve acquisition. Rade *et al.* estimated that a 60x improvement in the time required to navigate between cells in a large sample was obtained using this model [33].

The papers described here address problems which prevent the clinical adoption of AFM as a diagnostic tool. The described ML algorithms through optimisation of data accrual and analysis can both expedite the use of AFM as a research tool and facilitate it’s adoption by non-expert users in the clinical setting. However, coupling approaches aimed at expediting AFM data collection and reducing the user training required with ML based analysis classifiers to reduce or eliminate data analysis steps will ultimately enable widespread clinical adoption of AFM as a diagnostic tool.

### 4.4 Benefits and limitations of ML in AFM analysis

ML has a unique role in AFM as, if used correctly, it can make AFM more accessible to the wider scientific and clinical communities. This can be complementary to its more common role in biomedical research, where it has been used as a method for innovating and evaluating novel biomarkers. ML algorithms are free of interobserver judgement, but it is worth emphasising that the outputs are only as good as the inputs. If the standard of any element of sample preparation, specimen scanning, or analysis calculations are compromised for a given sample, then the results obtained are not fully comparable. This requirement to standardise sample handling is not unique to AFM and is equally a problem in the application of ML to pathology. However, the intricacies of model training provide multiple opportunities for a human to inadvertently alter the results. As is often the case in a new area, most studies apply independent methodologies and approaches, and few can be compared directly. This issue was previously noted by Albaradei *et al.* in their review of ML based analysis of omics data to predict metastasis [128]. While reproducibility is a concern, the NNs used in the majority of the above-described studies include training and account for generalisation in their design which should attend to those concerns. Indeed, models created with these deep learning methods have been shown to outperform less advanced models [128], [129]. However, it is pertinent to reiterate the necessity of reporting complete descriptions of validation and testing approaches.

## 5 AFM integration with diverse research techniques

As described above, much of the machine learning applied to AFM research has had either the explicit or resultant effect of speeding up AFM, often through automated analysis [111], [112], [114], [115]. It is clear that AI has the ability to progress AFM, but its coupling with other well-established techniques is becoming increasingly common. Fluorescence microscopy [107], immunohistochemistry [4], Raman spectroscopy [130], [131], and infrared spectroscopy [132], [133] have all been performed in conjunction with AFM. As a result, multiple datasets are obtained at both the same time and in the same location, allowing for enhanced spatial omic analysis. Combining spatial mechanical characterisation with spatial transcriptomics approaches [134], [135] provides the opportunity to further our understanding of disease and develop novel multi-modal biomarkers, and AI can facilitate integration of these multi-omic datasets. The potential benefits and pitfalls of ML when applied to multi-omic datasets are described in detail by Mohammed *et al*. [136]. Cost, time, noise, and choice of both model and input features are some of the greatest challenges faced when attempting such data heavy analyses [136]. Picard *et al.* noted that more is not always better when adding multi-omic datasets; including additional omic data is only appropriate if it is done correctly [137].

## 6 Conclusion

AFM offers detailed and accurate investigations of cells and tissues at the cellular length scale. It remains, however, most commonly used outside of biology. It has become an investigation of choice in a broad range of fields from semiconductors to materials science [138], [139]. Until recently, advances in AFM use in biological and cancer research have depended on knock on effects from these industries, for example integrated software programmes where force curves can be both acquired and analysed. Consequently, commercial upgrades to speed up AFM force-indentation curve acquisition have not always been relevant to, or useful in, biological research. Over the past decade, AFM research has matured and has clearly shown how biomechanical markers are present in cancer. Recently, systems exclusively made for biology are becoming more common and it is no longer a truly novel technique. Despite these recent improvements, significant impediments persist regarding many aspects of AFM, e.g. cost, physical space, and analysis expertise and time. AI can remedy many, but not all, of these challenges. The potential of AI has been successfully harnessed to accelerate both the acquisition and analysis of AFM measurements. However, the adoption of any specific ML algorithm as standard practice in AFM analysis is still some way off, nor is there any standardisation between the studies published in this field to date. Despite the problems described, AFM based nanomechanics and morphology data can provide for personalised diagnosis and prognosis and improved clinical decision making, leading to improved patient outcomes in cancer and other disease scenarios.

## Funding

This work was supported by St Vincent’s Foundation and the Irish Research Council Enterprise Partnership Scheme with Breakthrough Cancer Research (EPSPG/2023/1553 and EPSPG/2024/2194).

## CRediT authorship contribution statement

**Brian J Rodriguez:** Writing – review & editing, Supervision. **Tom K Gallagher:** Writing – review & editing, Supervision. **Aidan T O’Dowling:** Writing – review & editing, Writing – original draft, Conceptualization. **Stephen D Thorpe:** Writing – review & editing, Supervision, Conceptualization.

## Declaration of Generative AI and AI-assisted technologies in the writing process

The authors did not use any generative AI tools in the writing process for this manuscript.

## Declaration of Competing Interest

The authors declare that they have no known competing financial interests or personal relationships that could have appeared to influence the work reported in this paper.
